# Small fiber neuropathy in epidermolysis bullosa simplex

**DOI:** 10.1016/j.jdcr.2024.03.013

**Published:** 2024-04-03

**Authors:** Shahab Babakoohi, Amber Sipe, Mani Zamanifekri, William D. Hunter

**Affiliations:** a Atrium Health Levine Cancer Institute, Wake Forest School of Medicine, Charlotte, North Carolina; b Caromont Neurology, Gastonia, North Carolina; c Biology College, Columbia, South Carolina; d Neuroscience & Spine Center of the Carolinas, Gastonia, North Carolina

**Keywords:** epidermolysis bullosa simplex, small fiber neuropathy

## Introduction

Epidermolysis bullosa (EB) comprises several mechanobullous disorders characterized by the formation of large blisters. The 3 main types of EB include dystrophic, junctional, and simplex, distinguished by the site of skin integrity loss within the upper dermis, dermo-epidermal junction, and epidermis, respectively. Epidermolysis bullosa simplex (EBS) is the most prevalent type. Localized EBS, formerly called Weber-Cockayne type, is the mildest variant among all forms.^1^

Small nerve fibers are unmyelinated sensory axons that extend from the dermal-epidermal junction to the surface. Symptoms of small-fiber neuropathy (SFN) typically include distal burning, pain, numbness, and paresthesia. In a study involving patients with pure SFN, skin biopsy served as a diagnostic tool in 88 percent of the cases.^2^

There is limited literature exploring the relationship between EBS and SFN. In this case report, we present a patient diagnosed with EBS who developed SFN later in life.

## Case

**Fig 1:**
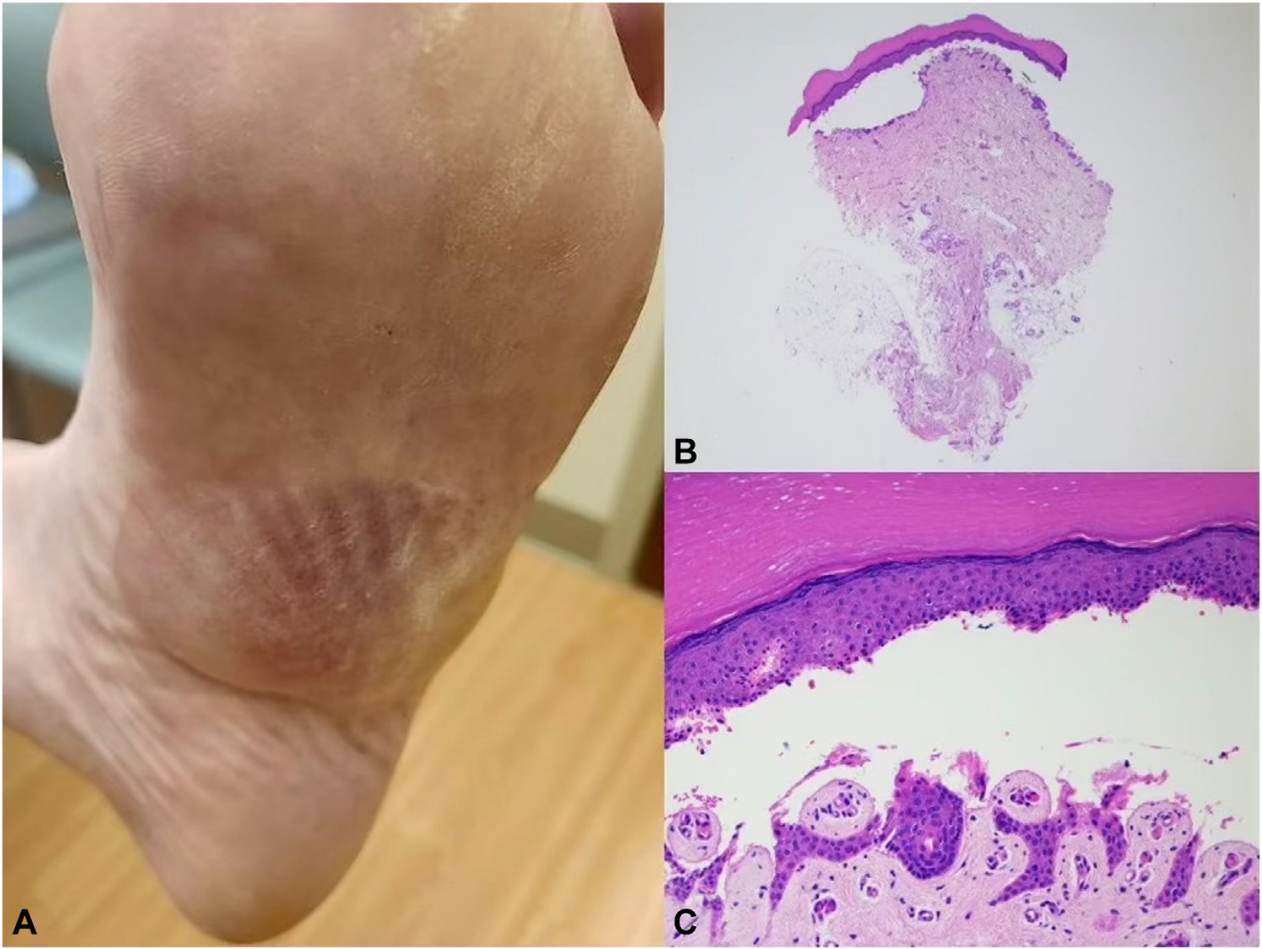
Large plantar blister elicited by heat. **A,** Histopathology of the cutaneous bulla showing intraepidermal blister (at the level of the lower to mid spinous layer) unaccompanied by significant inflammation either in the blister or in the subjacent dermis (**B**:10×, **C**:40×).

**Fig 2:**
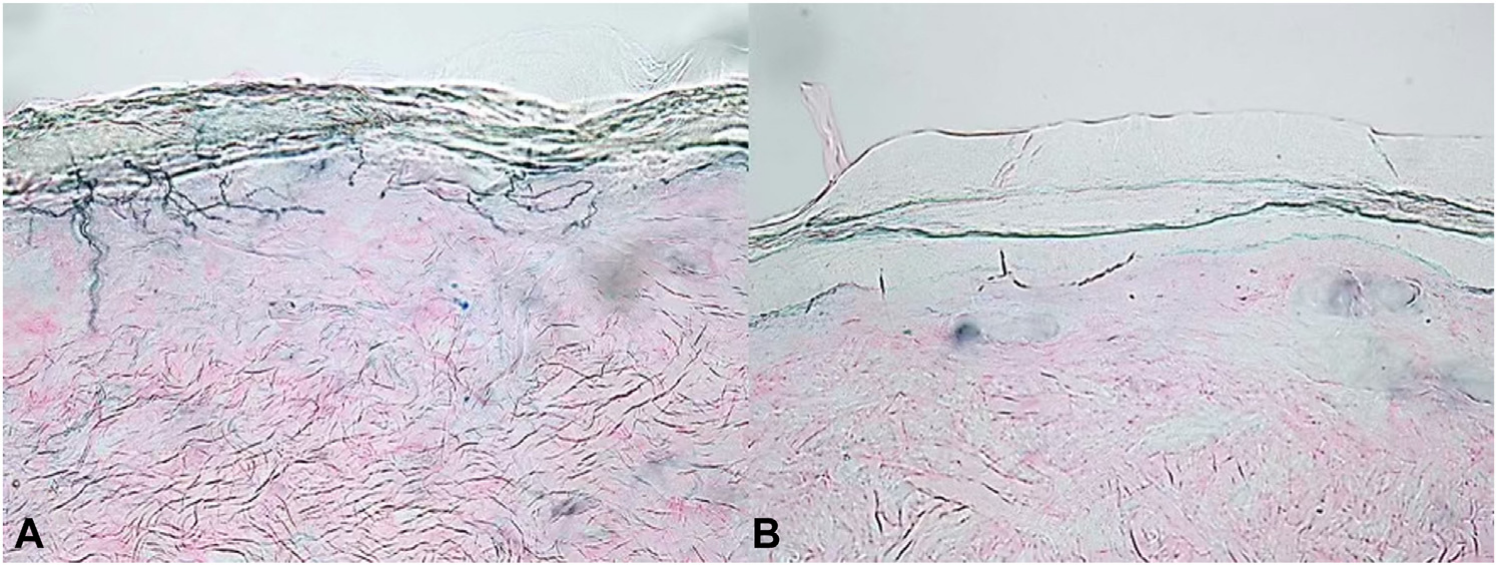
Immunostaining with an antibody to the axonal marker protein gene product-9.5 shows normal epidermal nerve fiber density of the small sensory nerve fibers in the skin of the thigh (**A**) and decreased epidermal nerve fiber density of these nerves in the skin of the ankle (**B**).

The patient, a 73-year-old man, had a known history of EB and presented with neuropathic pain symptoms such as tingling, pricking, and a burning sensation in both feet. Several members of his extended family, including his mother and 4 brothers, had a history of EB. EB was diagnosed during childhood based on clinical evaluation and medical history. His blistering episodes primarily affected his hands and feet, predominantly during the summer months (Fig 1, *A*). He coped with the condition by employing conservative measures, such as frequent application of emollients, wearing comfortable footwear, and minimizing perspiration, during the summer. Painful paresthesia emerged approximately 15 years before his consultation. An electromyography study of both lower extremities at the time of presentation revealed no signs of large-fiber neuropathy. Vitamin B12, thyroid-stimulating hormone, serum protein electrophoresis, creatine phosphokinase, folic acid, vitamin B1, antinuclear antibody, erythrocyte sedimentation rate, complete blood count, comprehensive metabolic panel, and hemoglobin A1c levels yielded unremarkable results. A skin biopsy of one of the active blisters yielded pathological findings consistent with EB (Fig 1, *B* and *C*). Another skin biopsy of the left lower leg above the lateral malleolus confirmed severe SFN (Fig 2, *A* and *B*). Subsequent, genetic testing for EB was conducted, revealing a heterozygous pathogenic variant of keratin (KRT)5, c.482T > G (p.Ile161Ser), consistent with an autosomal dominant pattern.

## Discussion

EBS is categorized based on suprabasal or basal epidermal cleavage, with localized EBS falling into the latter category. Localized EBS, the mildest form, presents with localized blisters confined to the palms and soles or other areas prone to trauma, often worsening during summer months.^3^ Our patient’s clinical presentation aligns closely with its description. Mutations in KRT5 and KRT14 genes contribute to approximately 75% of EB cases.^4^

SFN is a form of peripheral neuropathy that affects small nerve endings in the epidermis, the same cutaneous layer involved in EBS. However, the correlation between mechanobullous disorders and SFN remains understudied, likely due to the rarity of both entities.

In 1 study, SFN was reported in the majority of patients with the severe form of EB, known as recessive dystrophic epidermolysis bullosa. The authors concluded that dermoepidermal junction derangement in recessive dystrophic epidermolysis bullosa leads to injury to the small nerve fibers, especially in long axons, resulting in neuropathic pain^5^; However; in most cases, the underlying etiology of recessive dystrophic epidermolysis bullosa is a mutation of the collagen type VII alpha 1 chain gene (COL7A1), which encodes a different protein from the keratins affected in EBS. The role of keratinocytes in nerve regeneration has also been investigated. In a rat model, human hair keratin promoted the regeneration of peripheral sciatic nerves and axonal extension.^6^ Several neuron-keratinocyte interactions with mutual stimulation have been recognized, including the mitogenic effects of neurotransmitters on keratinocytes, nerve growth factor production by keratinocytes, and keratinocyte proliferation stimulated by neurotrophin-3 stimulation.^7^ Autoimmunity has been postulated to be the underlying etiology of SFN. In a study of 58 patients with SFN, several autoantibodies were higher in affected individuals than in healthy controls. Indeed, the KRT8 autoantibody was one of the 3 proteins, along with MX dynamin like GTPase 1 and Drebrin like-which, exhibited consistently high fold changes in patients with SFN with statistical significance.^8^ This raises the prospect of investigating autoantibodies against mutated keratins (KRT5/KRT14) implicated in EBS ^1^ in patients with SFN to ascertain whether these proteins play a role in the neurodegeneration process.

To the best of our knowledge, this is the first reported case of concurrent EBS and SFN. This correlation did not prove causation. Given the mild and frequent seasonal nature of EBS, it is conceivable that this underlying dermatological disorder may be overlooked in some patients with SFN. More retrospective studies from bullous disorder referral centers are required to investigate the prevalence of SFN in these patients. From a clinical perspective, it may be prudent to intensify skin care in patients with EBS who are in the early stages of SFN. This approach aims to mitigate excessive keratinocyte injury, thereby potentially impeding the development and progression of SFN.

## Conflicts of interest

None disclosed.
